# Transient abnormal acylcarnitine profile in newborn screening mimicking multiple acyl-Coenzyme A dehydrogenase deficiency associated with maternal sertraline use

**DOI:** 10.1016/j.ymgmr.2026.101319

**Published:** 2026-05-17

**Authors:** Rebecca Barbetti, Kate Coleman, Louise Fraser, Kalliope Demetriou, Elaine Jayadiwangsa, Drago Bratkovic, Carol Wai-Kwan Siu

**Affiliations:** a South Australia Neonatal Screening Centre, SA Pathology at Women's and Children's Hospital, North Adelaide, South Australia 5006, Australia; b Metabolic Unit, Women's and Children's Hospital, North Adelaide, South Australia 5006, Australia; c Adelaide Medical School, Faculty of Health and Medical Sciences, The University of Adelaide, Adelaide, SA 5000, Australia

**Keywords:** Multiple acyl-Coenzyme A dehydrogenase deficiency (MADD), Glutaric aciduria type II (GAII), Sertraline, Neonatal, Newborn, Screening, Acylcarnitine profile (ACP)

## Abstract

**Background:**

Multiple acyl-Coenzyme A dehydrogenase deficiency (MADD) is an inborn error of metabolism affecting fatty acid, amino acid and choline oxidation and is included in newborn screening in Australia. Recent reports describe adults with clinical and biochemical features of MADD, but negative genetic findings, associated with sertraline use.

**Aim:**

To describe a well newborn found to have a transient MADD-like biochemical pattern on newborn screen analysis, attributed to maternal sertraline use.

**Results:**

The newborn was delivered via semi elective C-section at term and was well at delivery, with age-appropriate growth percentiles. The mother had been taking sertraline throughout pregnancy and at delivery. Newborn screen analysis on day 2 showed elevation in multiple acylcarnitines (C5DC, C6, C8, C10 and C14:1), suggestive of MADD. The neonate remained well post-delivery and blood glucose and lactate levels at 1 and 4 h of age were normal.

Repeat acylcarnitine profile on day 5 of life showed improving values. Urine organic acids showed mild elevation of 2-hydroxyglutarate, supporting the MADD-like biochemical pattern. By day 12 of life, the acylcarnitines had normalised. Extensive gene panel analysis, including ETFA, ETFB, and ETFDH, identified no variants of clinical significance.

**Conclusion:**

Maternal sertraline use is a potential cause of a transient neonatal MADD-like biochemical pattern. Such medication-related effects should be considered when reviewing the possible aetiology of newborn screen results suggestive of MADD.

## 1 Introduction

Newborn Screening (NBS) protocols enable the early identification of treatable conditions which may have an initial asymptomatic period. Early detection and intervention can significantly modify the natural course of these conditions. NBS programs in Australia include acylcarnitine profiles which serve to detect inborn errors of fatty acid and organic acid metabolism.

Following a positive screen result, second tier investigations help to support the diagnosis, with associated pathogenic monogenic variants providing diagnostic confirmation.

Multiple acyl-Coenzyme A dehydrogenase deficiency (MADD; OMIM 231680), also known as glutaric aciduria type II (GAII), is an autosomal recessive condition that may be detected with NBS. Classical MADD is caused by biallelic pathogenic variants in the electron transfer flavoprotein alpha subunit (*ETFA*), electron transfer flavoprotein beta subunit (*ETFB*) or electron transfer flavoprotein dehydrogenase (*ETFDH*) genes. MADD can manifest with a spectrum of clinical presentations, including severe early neonatal onset, with or without congenital anomalies, and a late-onset presentation, which usually has a milder associated phenotype [1]. Pathogenic variants in genes involved in riboflavin metabolism have also been implicated [1].

Recent publications have described adults where MADD has been diagnosed clinically and biochemically, but for whom no genetic cause was identified [2], [3], [4], [5]. In several cases, an association with the use of sertraline was identified. There is some suggestion that the clinical and biochemical features of MADD may improve upon cessation of sertraline.

This case report serves to provide a reference for clinicians to consider maternal sertraline use as a cause for a transient MADD-like biochemical pattern on NBS results, with no confirmatory genetic findings.

## 2 Case report

The neonate was delivered via semi-elective caesarean section at 38 weeks and 2 days gestation, due to maternal preeclampsia. The neonate was well on delivery, with APGAR scores of 9 and 9. Vital signs were within normal limits. Blood glucose levels and lactate at 1 h and 4 h were all within normal limits.

The newborn screening results on the day 2 dried blood spot (DBS) demonstrated an abnormal acylcarnitine profile (ACP) with a pattern suggestive of MADD, characterised by elevations in medium-chain acylcarnitines, including octanoyl-carnitine (C8), long-chain acylcarnitines, such as tetradecenoyl-carnitine (C14:1), as well as glutaryl-carnitine (C5DC) (see Table 1 for other results). The abnormal NBS results were referred to the Metabolic Consultant when the neonate was 5 days of age. Full examination by the Neonatologist was normal – no phenotypic findings were identified. Blood gas, ammonia, and liver function tests were not concerning. Both maternal and neonate riboflavin levels were taken before replacement commenced. Riboflavin levels of the neonate were normal, while maternal riboflavin was 104 nmol/L (reference range 174–471 nmol/L.

**Table 1:** Newborn screening test results – tandem mass spectrometry analysis.

Acylcarnitine	Result	Reference interval
Carnitine (C0)	35 umol/L whole blood	15–73
Glutaryl-carnitine (C5DC)	0.27 umol/L whole blood	0.03–0.11
C5DC to C4 ratio	0.80	0.09–0.57
C5DC to C8 ratio	0.22	0.5–1.5
C5DC to C12 ratio	0.10	0.22–0.75
Hexanoyl-carnitine (C6)	0.38 umol/L whole blood	0.03–0.11
Octanoyl-carnitine (C8)	1.23 umol/L whole blood	0.03–0.15
Decanoyl-carnitine (C10)	2.31 umol/L whole blood	0.03–0.22
Decenoyl Carnitine (C10:1)	0.22 umol/L whole blood	0.04–0.15
C8 to C10 ratio	0.53	0.48–1.12
C8 to C6 ratio	3.25	0.67–1.83
Dodecanoyl Carnitine (C12)	2.63 umol/L whole blood	0.06–0.33
Dodecenoyl-carnitine (C12:1)	1.08 umol/L whole blood	0.02–0.25
Tetradecanoyl-carnitine (C14)	1.74 umol/L whole blood	0.11–0.49
Tetradecenoyl-carnitine (C14:1)	1.91 umol/L whole blood	0.06–0.37
Tetradecadienoyl-carnitine (C14:2)	0.22 umol/L whole blood	0.03–0.1
C14:1 to C4 ratio	5.60	0.1–1.55
C14:1 to C10 ratio	0.83	0.82–3
C14:1 to C12:1 ratio	1.64	1.1–3.33

Repeat ACP testing on DBS on day 5 showed less pronounced acylcarnitine elevations but still indicative of MADD. Urine organic acid results demonstrated a mild increase in 2-hydroxyglutarate (2-OH glutarate), which supports the ACP findings suggesting MADD [1], [2], [3].

On day 6 of life, the neonate was commenced on riboflavin 100 mg/day and 3 hourly feeds, while awaiting genetic testing results.

Further repeat ACP DBS testing on days 12 and 39 of life were normal. Urine organic acids were not repeated.

Genetic testing results were returned on day 27 and did not determine a genetic cause. Genetic testing was performed as per Fatty Acid Oxidation Defects v.1.14 (PanelAppAus, green and amber genes) [6] and Undiagnosed metabolic disorders v.1.924 (PanelAppUK, green genes) [7] with the addition of SLC52A1 at the request of the clinician. Results reported that sequence analysis had not detected any pathogenic sequence variation. No deletions/duplications were detected.

Review of the clinical history revealed that the mother had been taking sertraline throughout pregnancy and at delivery. The mother's sertraline dose at the time of delivery was 100 mg/day.

On day 39 of life, the neonate remained asymptomatic, repeat ACP was normal, riboflavin was ceased and the neonate was discharged from the metabolic clinic with the diagnosis of no MADD, with previous ACP and urine organic acid results being attributed to maternal sertraline use.

Repeat riboflavin in the mother 18 months post-delivery was within normal range at 216 nmol/L. At the time of repeat riboflavin testing, the mother was still taking sertraline (50 mg/day) and was not taking any riboflavin replacement. Maternal ACP and urine organic acids were also performed at the time of repeat riboflavin testing. Maternal ACP was normal and maternal urine organic acids showed unspecific changes. The mother did not exhibit any clinical signs of MADD.

Results are summarised in Table 2.

**Table 2:** Results.

Examination	No abnormal findings
Blood glucose 1 h	3.9 mmol/L
Blood glucose 4 h	4.0 mmol/L
Lactate 1 h	2.2 mmol/L
Lactate 4 h	1.4 mmol/L

Capillary blood gas Day 5:	
pH	7.42
pCO2	39 mmHg
pO2	36 mmHg
HCO3^−^	25 mmol/L
Base Excess	0.6 mmol/L

Ammonia	69 umol/L (≤ 99)
Liver Function Tests Day 5:	
GGT	381 U/L (0–140)
ALP	21 U/L (80–380)
ALT	28 U/L (0–35)
AST	70 U/L (0–50)

Neonatal riboflavin (prior to supplementation)	241 nmol/L (174–471)
Maternal riboflavin (at delivery)	104 nmol/L (174–471)
Maternal riboflavin (18 months post-delivery)	216 nmol/L (174–471)

Urine organic acids	Mild increase in 2-hydroxyglutarate.

Renal ultrasound	No structural renal abnormality detected

Genetic testing	No variants of clinical significance detected

## 3 Discussion

### 3.1 Overview of literature describing association of MADD with sertraline use in adults

It is well-established that maternal sertraline crosses the placenta during pregnancy [8]. Furthermore, multiple recent publications have identified an apparent correlation between sertraline use and diagnosis of late-onset MADD (in adults). Sunebo et al. published a report describing 9 patients diagnosed with late-onset MADD [2]. A probable genetic cause was identified in 2 of these patients. Of the remaining 7 patients, all were taking sertraline at the onset of symptoms. Demetriou et al. [3] performed a molecular genetic analysis of candidate genes for MADD for 28 patients (18 adult and 10 paediatric) with a MADD diagnosis based on clinical and biochemical findings. Of the 13 adult patients for whom a molecular variant for MADD was not identified, 7 were taking sertraline. Another one adult patient with no identified molecular variant was taking the serotonin-reuptake inhibitor (SNRI) duloxetine, which has a similar mechanism of action to sertraline. 3 patients with monoallelic variants were also taking either serotonin or venlafaxine, another SNRI. Ingoglia et al. [4] identified 2 patients with MADD-like patterns of ACP associated with sertraline use, with additional investigations supporting a possible deficit in electron transport chain function. A case study published by Gaini et al. [5] described a 72-year-old woman on sertraline who was diagnosed with very-late-onset riboflavin-responsive MADD. Overall, there is increasing evidence suggesting a correlation between sertraline use, and what appears to be an acquired form of MADD.

### 3.2 Plausible mechanism for association of MADD with maternal sertraline use

There have been several reports suggesting a possible effect of maternal sertraline on the developing fetus. A study investigating the effect of maternal SSRI intake on fetal cardiac function by Shen et al. [9] observed that SSRI exposure inhibited ATP production and mitochondrial respiration and disrupted mitochondrial homeostasis in cardiomyocytes. Another recent study investigating the relationship between maternal sertraline intake and congenital heart defects by Lu et al. [10] used a mouse model of in utero plus neonatal sertraline exposure and observed a significant upregulation of specific microRNAs that modulate serotonin signalling in neonatal cardiac tissues, with corresponding levels of the target RNAs downregulated, resulting in decreased production of the proteins.

Yan Li et al. [11] investigated mitochondrial dysfunction induced by sertraline, in the context of developing a possible mechanism for liver injury induced by sertraline. In this study, it was found that, in mitochondria isolated from rat liver, sertraline uncoupled mitochondrial oxidative phosphorylation and inhibited the activities of oxidative phosphorylation complexes I and V.

Harmonizome 3.0 lists miR25-3p as a microRNA targeting the ETFA gene in low- or high-throughput microRNA targeting studies from the MiRTarBase microRNA Targets dataset [12] [13] [14]. MiR25-3p has been identified as being significantly upregulated after sertraline treatment [15]. These findings, considered in parallel with that reported by Lu et al. [10] whereby the upregulation of microRNAs corresponded to downregulation of target RNAs and decreased production of cognate proteins, demonstrates a plausible link connecting sertraline use with ETFA levels which would therefore explain a possible mechanism for maternal sertraline use producing the neonatal acylcarnitine profile seen in the case presented here.

### 3.3 Sertraline and breastfeeding

It is worthwhile considering any possible contribution of sertraline in breastmilk as a factor in the biochemical findings of MADD in this patient. Sertraline is also known to be present in breastmilk in small amounts. Current evidence suggests that the ingestion of sertraline by neonates via breastmilk is low at best [16], although this is not confirmed.

As documented in the clinical notes, the neonate was breastfeeding up until at least 4 months of age. The last abnormal ACP was collected on day 5 of life, and the first normal ACP was collected on day 12 of life. The mother was an inpatient for 10 days after delivery and no sertraline was charted during this time. Therefore, it can be concluded that breastfeeding whilst taking sertraline is unlikely to have contributed to a MADD-like biochemical profile in this case.

### 3.4 Low maternal riboflavin as possible confounding result

Cases in the literature describe a possible relationship between low maternal riboflavin and MADD-like biochemical findings in the neonate [17] [18]. Hagemeijer et al. [17] reported on 4 cases of newborn screening results with acylcarnitine profiles resembling MADD. In each of these cases, the newborns were found to have low riboflavin deficiency. It was hypothesised that the low riboflavin levels in the newborns were due to maternal riboflavin deficiency. However, maternal riboflavin was not tested in any of these cases. In addition, the newborn in the case reported here had a normal riboflavin (see results in Table 2). Chiong et al. [18] reported a newborn presenting with clinical and biochemical findings consistent with MADD. The baby's riboflavin levels were never tested, although biochemical findings (urine organic acids and acylcarnitine profile) improved with riboflavin replacement of 100 mg/day. The mother was found to be riboflavin deficient post-delivery and remained riboflavin deficient in repeat testing 2 years later. It was postulated that a primary genetic defect in riboflavin metabolism in the mother was the cause of the transient MADD in the neonate (although this was not confirmed with genetic studies). Conversely, in the case presented here, the mother was riboflavin replete in repeat testing 18 months post-delivery without riboflavin replacement, thus making a maternal genetic defect in riboflavin metabolism is unlikely.

## 4 Conclusion

This report describes an asymptomatic neonate with a newborn screen result suggestive of multiple acyl-Coenzyme A dehydrogenase deficiency (MADD). The urine organic acid profile supported the acylcarnitine results, which subsequently normalised within 6 weeks of birth. Genetic testing did not identify any diagnostic pathogenic variants. A significant association was of maternal sertraline use during pregnancy.

There is currently no published literature showing a correlation between maternal sertraline use and a MADD-like biochemical pattern on NBS. However, recent reports have suggested a correlation between sertraline use and a MADD-like clinical and biochemical phenotype in adults.

A possible mechanism for these biochemical findings in newborns is that sertraline may be impacting mitochondrial function in the fetus.

In conclusion, maternal sertraline use should be considered as a possible cause for a transient MADD-like pattern on NBS, with normal molecular investigations.

## CRediT authorship contribution statement

**Rebecca Barbetti:** Writing – review & editing, Writing – original draft, Investigation. **Kate Coleman:** Writing – review & editing, Investigation. **Louise Fraser:** Writing – review & editing, Investigation. **Kalliope Demetriou:** Writing – review & editing, Investigation. **Elaine Jayadiwangsa:** Writing – review & editing, Investigation. **Drago Bratkovic:** Writing – review & editing, Investigation. **Carol Wai-Kwan Siu:** Writing – review & editing, Investigation.

## Declaration of competing interest

None.

## Data Availability

No data was used for the research described in the article.
